# A hybrid approach based on multipath Swin transformer and ConvMixer for white blood cells classification

**DOI:** 10.1007/s13755-024-00291-w

**Published:** 2024-04-28

**Authors:** Hüseyin Üzen, Hüseyin Fırat

**Affiliations:** 1 Department of Computer Engineering, Faculty of Engineering and Architecture, Bingol University, Bingol, Turkey; 2 Department of Computer Engineering, Faculty of Engineering, Dicle University, Diyarbakır, Turkey

**Keywords:** White blood cell classification, Deep learning, Swin transformer, ConvMixer, Multipath mixer

## Abstract

White blood cells (WBC) play an effective role in the body’s defense against parasites, viruses, and bacteria in the human body. Also, WBCs are categorized based on their morphological structures into various subgroups. The number of these WBC types in the blood of non-diseased and diseased people is different. Thus, the study of WBC classification is quite significant for medical diagnosis. Due to the widespread use of deep learning in medical image analysis in recent years, it has also been used in WBC classification. Moreover, the ConvMixer and Swin transformer models, recently introduced, have garnered significant success by attaining efficient long contextual characteristics. Based on this, a new multipath hybrid network is proposed for WBC classification by using ConvMixer and Swin transformer. This proposed model is called Swin Transformer and ConvMixer based Multipath mixer (SC-MP-Mixer). In the SC-MP-Mixer model, firstly, features with strong spatial details are extracted with the ConvMixer. Then Swin transformer effectively handle these features with self-attention mechanism. In addition, the ConvMixer and Swin transformer blocks consist of a multipath structure to obtain better patch representations in the SC-MP-Mixer. To test the performance of the SC-MP-Mixer, experiments were performed on three WBC datasets with 4 (BCCD), 8 (PBC) and 5 (Raabin) classes. The experimental studies resulted in an accuracy of 99.65% for PBC, 98.68% for Raabin, and 95.66% for BCCD. When compared with the studies in the literature and the state-of-the-art models, it was seen that the SC-MP-Mixer had more effective classification results.

## Introduction

Blood cells mainly consist of red blood cells, platelets and white blood cells (WBCs) [1]. Of these blood cells, WBCs are primarily responsible for the defense of the human body. They play an active role in the immune system because it protects the human body against various microorganisms such as parasites, viruses and bacteria [2]. WBCs are classified into distinct subgroups based on their morphological configurations. These subgroups encompass Neutrophils, Eosinophils, Basophils, Lymphocytes, Monocytes, Platelets, Erythroblasts and immature granulocytes such as promyelocytes, myelocytes, and metamyelocytes [3]. Each of these subgroups has the role of defending the body against foreign pathogens. Hence, the count of different WBC subgroups offers substantial diagnostic insights into conditions like leukemia, AIDS, blood cancer, and anemia [4]. Accurate identification of the appropriate WBC holds considerable clinical importance. The primary responsibility in this realm lies in categorizing WBCs within the bloodstream. However, due to the morphological differences in the images of WBC subtypes, it is difficult to classify WBC images into subtypes. Initially, classification of WBC images into subtypes was performed with a hematology analyzer with the help of a specialist hematologist. However, since this process is carried out manually, it is very time consuming and can result in misclassification [5]. Lately, within the realm of medical image analysis, there has been a frequent utilization of deep learning (DL), notably methods based on Convolutional Neural Networks (CNNs) [6, 7]. DL enhances classification accuracy through the automated extraction of features [8]. CNN’s robust self-learning abilities enable the extraction of more profound features with richer semantic content from images [9, 10]. Recently, studies incorporating CNN-based approaches for WBC classification have emerged and gained widespread use, diverging from traditional classifiers. However, while CNN-based approaches have achieved success in image classification, they heavily rely on local receptive fields and pooling operations. They may struggle with the high intra-class variability and small object sizes in WBC images. Moreover, their focus on local features (or receptive fields) may limit their ability to capture long-range contextual information, crucial for distinguishing subtle morphological variations among WBC types. This limitation hinders their potential to comprehensively understand the input and grasp complex relationships between various regions of the image. Additionally, CNN architectures often require meticulous engineering and optimization efforts to achieve optimal performance on specific datasets, thereby reducing their flexibility and adaptability. Unlike traditional CNNs that use spatial convolutions to extract features from images, the Swin Transformer model, employing self-attention mechanisms to capture relationships between different regions of an image, can enhance performance. Additionally, ConvMixer can effectively extract local or spatial features from WBC images due to its convolutional layers. Based on this, a hybrid approach that uses ConvMixer and Swin transformer models together is proposed in this study.

The Swin transformer and Vision Transformer (ViT) models, recently introduced, have seen widespread application across numerous studies within the domain of medical image analysis [11–13]. These structures break down the input and convert it into tokens. Then, these tokens are transformed into powerful long contextual features with their self-attention mechanism. While the self-attention mechanism is quite effective in extracting long context information, transformer blocks lose spatial details [14, 15]. In this context, some studies have used transformer blocks in the last layers of network architectures [16, 17]. There are many benefits to adding transformer blocks to the final layers of network architectures. Transformers are adept at capturing long-range contextual information within data [18]. But are poor at capturing spatial details such as color and texture [19]. In addition, in traditional CNNs, strong spatial details are obtained in the first layers while strong semantic features are obtained in the last layers [19, 20]. By adding transformers to the last layer, the model can learn more complex relationships between different parts of the image. Therefore, it can obtain more effective global features by processing dense semantic features in the last layer of network architectures. In addition, Transformers focus on specific and relevant features in the processed data [21]. Therefore, giving strong initial features to transformer models facilitates the training of weights in the self-attention mechanism. Finally, transformers obtain the output by associating all tokens with the self-attention mechanism they contain. Therefore, a large number of tokens increases the transaction cost exponentially [22]. Therefore, the lower spectral size obtained at the end of the network can reduce the transaction cost by obtaining a smaller number of tokens. On the other hand, Trockman et al.[22], considering that the main source of success of transformer models is fragmentation, presented a new and effective structure with low parameters, called ConvMixer. ConvMixer is basically inspired by transformer models and aims to extract effective features by segmenting the image. Additionally, ConvMixer blocks use depth-separable convolution instead of the self-attention mechanism which has high processing costs. Thanks to this structure, it enables to obtain stronger spatial features from the input image compared to transformers. In addition, they argue [22] that different part sizes and core sizes have an impact on performance. Although depth-separable convolution layers are an efficient structure with low processing costs, they cannot extract global features by directly associating part tokens as in the self-attention mechanism. Based on these, a new hybrid network architecture was developed using ConvMixer and Swin transformer structures in this study to classify WBCs. In this model, called Swin transformer and ConvMixer based Multipath mixer (SC-MP Mixer), ConvMixer blocks extract features with strong spatial detail, while Swin transformer networks effectively process these features with self-attention mechanism. Also, the proposed model uses a multipath approach to obtain better patch representations for ConvMixer and Swin transformer blocks. In this approach, ConvMixer and Swin transformer blocks are applied in parallel with different patch sizes. In this way, more effective features can be obtained with different patch representations. In conclusion, our study’s primary contributions can be outlined as follows:

1. A new network architecture is designed by combining ConvMixer and Swin transformer structures in the SC-MP-Mixer model.
2. Stronger spatial and long-context informaton is obtained by using different patch representations in the proposed model.
3. In experimental studies on three different WBC datasets, the SC-MP-Mixer model has achieved high success against the state-of-the-art models.

The rest of this study is structured accordingly. “Related works” Sect. contains related literature, encompassing recent studies. “Material and method”, Sect. titled Material and Methods, elaborates on the datasets utilized in the experiments and presents information about the SC-MP-Mixer model. “Experimental studies and results” Sect. will cover the experiments conducted and their outcomes. Lastly, “Conclusions” Sect. serves as the conclusion, summarizing our findings and considering future avenues.

## Related works

In recent years, there have been numerous studies proposing DL approaches for WBC image analysis in the field of microscobic blood cells. One of the common objectives of these studies is to automate the diagnosis of blood diseases using WBC images, which can improve diagnostic accuracy and reduce the workload of hematologists. Recent studies of blood diseases abnormalities with WBC images have shown that manual evaluation of multiple WBC images is laborious and requires expertise. In this direction, efficient intelligent DL, especially CNN-based methods have been developed to assist hematologists in their tasks. Thanks to these methods, correct treatment recommendations are shown by automatically extracting the image features through convolutions, processing and analyzing the image data. Also, the use of CNN-based methods showed better classification performance in feature extraction, making them cutting edge for deep learning applications. Efficient use of CNN has developed tasks related to image classification and recognition. Some of the studies using CNN in the literature are given below.

Shahin et al. [23] introduced a novel approach, WBCsNet, for WBC classification, employing a deep CNN architecture. This method integrates three convolutional layers, two pooling operations, four ReLUs, two fully connected (FC), and Softmax layers. To assess WBCsNet’s classification efficacy, experiments were conducted using a dataset comprising 2551 images featuring 5 distinct WBC types. The experimental findings revealed a classification accuracy of 96.1%. Bani-Hani et al. [24] applied a CNN approach to categorize four categories of WBC images: eosinophils, neutrophils, lymphocytes and monocytes. Additionally, they employed the genetic algorithm to optimize the hyperparameters within the CNN methodology. Upon analyzing the results from the BCCD dataset, an overall accuracy of 91.01% was achieved. Tiwari et al. [25] created a novel CNN structure for WBC classification. The approach integrates two convolution, pooling, FC, and classification layers. After applying this method to a dataset containing around 13,000 WBC images, a classification accuracy of 78% was achieved for four distinct WBC types. Sharma et al. [26] performed experiments employing the LeNet5 model to classify WBCs. The experimental investigations on the BCCD dataset, consisting of four classes, resulted in an accuracy of 87.93%. Banik et al. [27] suggested a CNN model designed for WBC classification, comprising five convolutional layers, three maximum pooling layers, and a FC layer. Within this CNN structure, the feature maps from two convolutional layers are merged using maximum pooling before being fed into the FC layer. To evaluate the CNN’s classification prowess, tests were conducted using the BCCD dataset, encompassing 4 WBC classes. The experimental analysis revealed a classification outcome of 90.79%. Yao et al. [28] introduced the weighted optimized deformable CNN method, comprising two modules, for WBC classification. Evaluating this method’s performance involved conducting experiments using the BCCD dataset, which encompasses four WBC classes. The experimental outcomes yielded values of 91.6% for precision, recall and F1-score. Sharma et al. [29] presented a fast traditional CNN method for WBC classification. This method consists of three convolution and three FC layers. However, each convolution layer includes ReLU, max-pooling and dropout layers. When the results of the experimental studies performed on the four-classes BCCD dataset are examined, it is seen that the classification accuracy of 84.64% was obtained. Uçar [30] suggested ShuffleNet architecture for WBC classification. With this method, as a result of the experimental studies carried out on the eight-class dataset, 97.94% classification accuracy was obtained.

In addition to studies using only CNN-based methods, another method used in the literature is the hybrid methods developed together with CNN for WBC classification. Patil et al. [1] devised a deep hybrid DL technique for WBC classification based on canonical correlation analysis (CCA), integrating LSTM and CNN. Through the CCA, the method extracts diverse, intersecting features from the input image, elevating its accuracy in comparison to similar DL approaches. The classification accuracy obtained from experiments conducted on the BCCD dataset stands at 95.89%. Baydilli et al. [31] introduced a technique known as capsule networks for categorizing WBCs into five different types. The capsule networks are fundamentally comprised of two main components: an encoder and a decoder. The role of the decoder is to reconstruct the image, whereas the encoder is tasked with extracting features from the image and performing classification. The accuracy of this classification was assessed using the LISC dataset, comprising 263 images of WBCs. The implementation of this method revealed an accuracy of 96.86%. Şengür et al. [32] introduced a hybrid approach for WBC classification, merging image processing techniques with DL. Following the application of diverse image processing methodologies (RGB to HSV transformation, conversion from color to grayscale, filtering and thresholding) on WBC images, the WBC classification employed the LSTM technique. Evaluating the performance of experiments on the BCCD dataset to assess the hybrid method’s classification, an accuracy of 92.89% was achieved. Ekiz et al. [33] developed a fusion of CNN and Support Vector Machine (SVM) to classify WBC images utilizing a BCCD dataset containing four distinct classes. Their approach led to an accuracy of 85.96%.

Some studies use only pre-trained architectures for WBC classification, while others create hybrid methods using pre-trained architectures and different techniques. Tseng et al. [34] used 10 different pre-trained CNN architectures to classify six WBC types (segmented neutrophil, banded neutrophil, metamyelocyte, myelocyte, promyelocyte, myeloblast) in a dataset of 26,050 WBC images. The accuracy values obtained as a result of experimental studies with these 10 different CNN architectures are as follows: DenseNet (85.7%), ResNeSt (88.2%), MobileNet (87.0%), InceptionV3 (85.7%), ResNeXt (87.9%), InceptionResNetV2 (87.0%), RegNetY (87.3%), RegNetZ-C (87.5%), RegNetZ-D (88.6%), ConvNeXt (88.0%). Liang et al. [35] a hybrid method for WBC classification, which is a combination of LSTM and pre-trained Xception architectures. To evaluate the classification efficacy of their proposed approach, experiments were conducted using the BCCD dataset, which encompasses 4 WBC classes. The experimental investigations yielded an average accuracy of 90.79%. Furthermore, within this study’s framework, classification accuracy outcomes on the same dataset were observed at 88.58% for LSTM + ResNet50 + InceptionV3, 89.38% for LSTM + ResNet50 and 87.45% for LSTM + InceptionV3. Yu et al. [36] devised an approach for WBC classification, amalgamating pre-trained Xception, VGG 16–19, InceptionV3, and ResNet50 architectures. This hybrid method underwent testing on a dataset comprising 2000 images featuring seven distinct WBC classes. The attained classification accuracy stood at 88.5%. Baby et al. [37] suggested a hybrid method consisting of a combination of SVM and pre-trained DL architectures for WBC classification. They used Xception, InceptionV3, MobileNetV2, DenseNet121 and ResNet50 as feature extractors. They also utilized the extra trees classifier as an intermediate step to select the most selective features. Finally, they utilized the multi-class SVM for classification. For the performance analysis of this hybrid method, they performed experiments on a dataset of 431 WBC images containing 4 classes. According to the experimental studies performed, the classification accuracy results obtained are as follows: ResNet50 + SVM (90.76%), DenseNet121 + SVM (72.3%), MobileNetV2 + SVM (87.69%), IncepitonV3 + SVM (76.92%), and Xception + SVM (70.26%).

Upon reviewing the literature, it is evident that CNN-based approaches, CNN-based hybrid methods, and pretrained models are frequently employed in WBC classification. Within the same class, WBCs may exhibit significant morphological differences in terms of size, shape, texture, and nucleus-cytoplasm ratio, which can be crucial for WBC classification. Primarily, CNNs that focus on local features may not effectively capture long-range dependencies among different image regions. These dependencies, critical for distinguishing subtle morphological variations that differentiate WBC types, may not be adequately addressed by CNN-based pretrained models or hybrid methods. While additional techniques may offer some improvements, the fundamental challenges of CNNs may persist. To address the importance of capturing long-range dependencies in WBC classification, this study proposes a method combining Swin Transformer with ConvMixer. The Swin Transformer component alleviates a significant limitation by capturing long-range dependencies among different image regions, enabling analysis of relationships between various parts of WBCs, such as the size and position of the nucleus relative to the cytoplasm. This allows the model to better understand the overall morphology of WBCs, leading to enhanced differentiation among cell types with subtle differences. Additionally, ConvMixer preserves the strength of CNNs in extracting local features from WBC images through its convolutional layers, effectively capturing tissues, shapes, and fundamental structures within WBCs. By combining ConvMixer and Swin Transformer, the model may learn more robust and transferable features even with limited training data. Leveraging both local features and long-term dependencies, the model can derive more informative representations from the data, potentially leading to better generalization capabilities. To assess the effectiveness of the proposed model, the BCCD, PBC, and Raabin blood cell datasets were utilized. In the BCCD dataset, to ensure a fair comparison, the original dataset’s training and test examples were used as is. However, for the PBC and Raabin datasets, they were split into 70% for training, 15% for testing, and 15% for validation. Through extensive experimental studies, accuracy values of 95.66, 99.65, and 98.68% were achieved for the BCCD, PBC, and Raabin datasets, respectively.

## Material and method

### Swin transformer (SwTrans)

Transformers have received a lot of attention in the field of natural language processing since they were first published [38, 39]. The highlighting feature of transformers is that they have a self-attention mechanism that examines the relationship between words. It basically examines the relationship between all words in the mechanism of self-attention. In this way, it treats the input as a whole. The Convolution layer in CNN architectures processes information as much as the filter size. This situation is defined as regionality (locality of convolution operations) in the literature. On the contrary, because it processes all the input as a whole in transformers, it obtains strong global semantic attributes (long-range contextual information) [39]. Dosovitskiy et al. [38] proposed the ViT model to adapt transformers to the field of computer vision (CV) in 2020. In the ViT model, tokens are obtained by first fragmenting the image. Then, global semantic details (long-range contextual information) were obtained by passing the tokens through the self-attention structure. In this way, unlike the convolution layers that deal with the relationship (filtering) between certain regions, strong features are obtained with this structure. With the development of the ViT model, transformers have been actively applied in the field of CV and have become quite common recently [14, 38, 40–42].

**Fig. 1:**
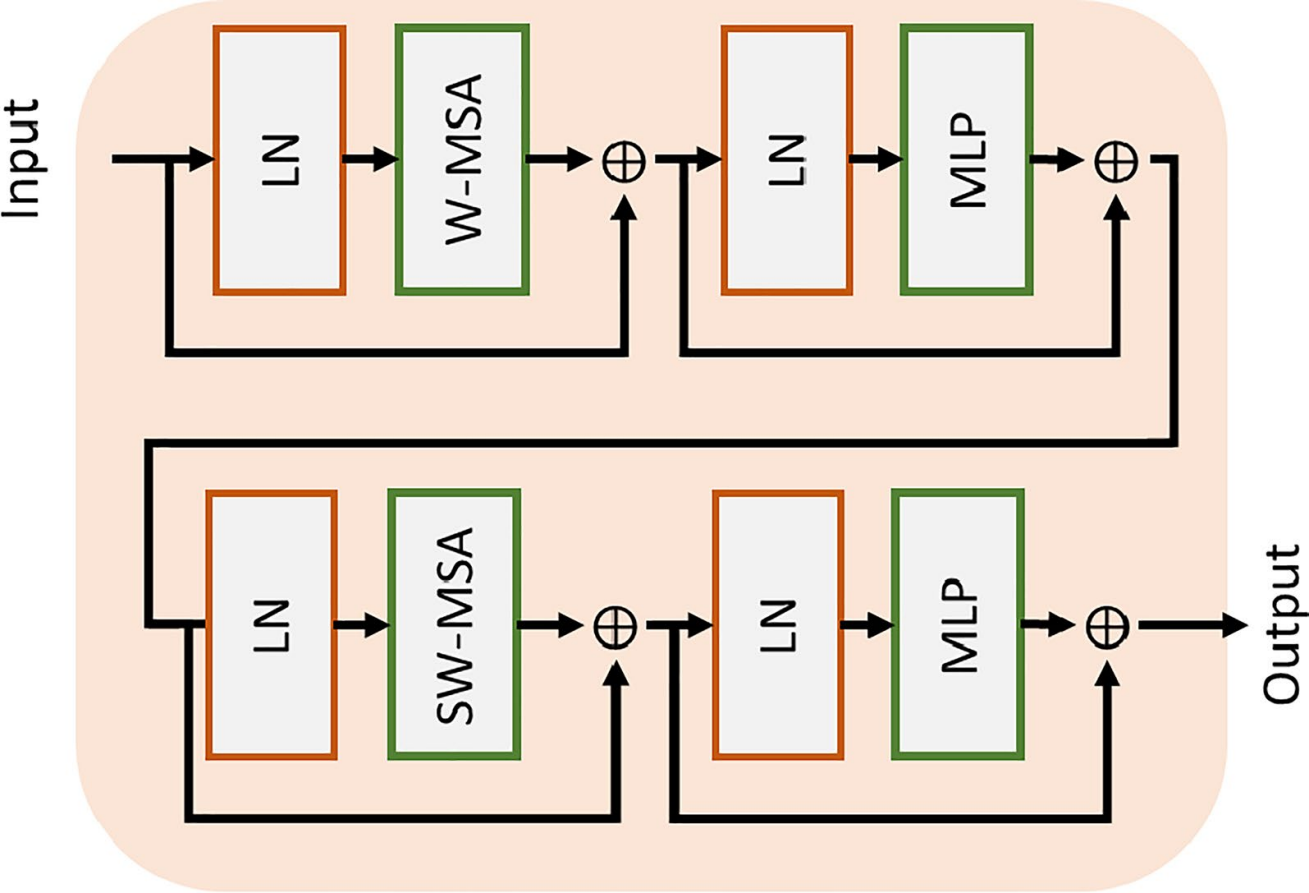
SwTrans BLOCK

Although the ViT transformer model gives successful results in image classification, it is time consuming and costly because all parts are associated with each other in the transformer model [16, 38]. It is also stated to be weak for CV problems such as detection and segmentation, as all parts are associated together [14, 15, 17]. The SwTrans (Fig. 1) is proposed to decrease the computational complexity of the ViT model and to exhibit a strong transformer structure for segmentation. In the SwTrans model, the local window model is used when evaluating the relationship between the parts. In this way, the self-attention mechanism was applied only to the parts inside the window instead of all the parts. Then, these windows are scrolled and the patches inside the window are changed and the process continues. For detailed information, see [14, 15, 17].

As can be seen in Fig. 1, the SwTrans model consists of two steps. In the first step, the parts inside the windows Window based MSA (W-MSA) module was applied. In the second step, the parts inside the window were changed by sliding the windows. After scrolling, the Shifted Window based MSA (SW-MSA) module is applied. In this way, the SwTrans model evaluates the relationship between the parts in different regions at a lower cost than the ViT model. SwTrans output (z) is calculated as in Eq. (1) [14]:

1 $$\begin{gathered} \hat{z}_{l} = WMSA\left( {LN\left( {z_{l - 1} } \right)} \right) + z_{l - 1} \hfill \\ z_{l} = MLP\left( {LN\left( {\hat{z}_{l} } \right)} \right) + \hat{z}_{l} \hfill \\ \hat{z}_{l + 1} = SWMSA\left( {LN\left( {z_{l} } \right)} \right) + z_{l} \hfill \\ z_{l + 1} = MLP\left( {LN\left( {\hat{z}_{l + 1} } \right)} \right) + \hat{z}_{l + 1} \hfill \\ \end{gathered}$$

In Eq. (1), $z_{l}$ and $z_{l + 1}$ denote the output feature vector of the WMSA and SWMSA, respectively. $MLP\left( . \right)$ represents multi-layer perceptron. In addition, the multi-layer perceptron function is basically the application of fully connected, GELU and dropout layers, respectively.

### ConvMixer

The ViT model has opened a new era in DL [41, 43]. However, the self-attention mechanism of the ViT model has second-order complexity. Therefore, the ViT model requires a high level of data and hardware requirements [22, 43]. Based on these problems, Trockman et al. [22] developed the ConMixer model by researching the performance source of the ViT model.

Trockman et al. thought that it could have a high success since it basically used a piece of image instead of pixels in the ViT model [22]. Starting from this point, he divided the input image into patches, as it did with the MLP mixer and the ViT model. It then applied a series of convolution operations to the output representing the patches. In the ConvMixer model, a convolution is used to split the image into patches. In this convolution layer, the kernel and step set the value to the patch size (p). This process is shown below:

2 $$z_{0} = BN\left( {\sigma \left( {Conv_{kernal:p}^{stride:p} \left( {image} \right) } \right)} \right)$$

The $z_{0}$ and $p$ given in Eq. (2) represent the output of splitting the image into patches and the patch size. As shown in Eq. (2), after the convolution process, GELU (σ) and Batch normalization (BN) layers were applied, respectively. In addition, the number of filters in the convolution operation is taken as $h$. As a result of Eq. (2) operations, an input image of $W \times H$ size and an output of $\frac{W}{p} \times \frac{H}{p} \times h$ size were obtained. Each array of pixels ($1 \times 1 \times h$) in this output represents different versions of a patch of size A.

ConvMixer has transferred the $z_{0}$ output obtained in Eq. (2) output to a depthwise separable convolution block. This convolution block is shown in Eq. (3).

3 $$\begin{gathered} z_{l}{\prime} = BN\left( {\sigma \left( {DepthWiseConv\left( {z_{l - 1} } \right)} \right)} \right) + z_{l - 1} \hfill \\ z_{l} = BN\left( {\sigma \left( {PointWiseConv\left( {z_{l}{\prime} } \right) } \right)} \right) \hfill \\ \end{gathered}$$

As shown in Eq. (3), the depthwise separable convolution block consists of two stages. These are, respectively, depthwise convolution and pointwise convolution. After each convolution operation, GELU (σ) and BN operations were implemented, respectively. Also, as shown in Eq. (2), the ConvMixer has Residual skip connections as in the ViT model.

### Proposed method

**Fig. 2:**
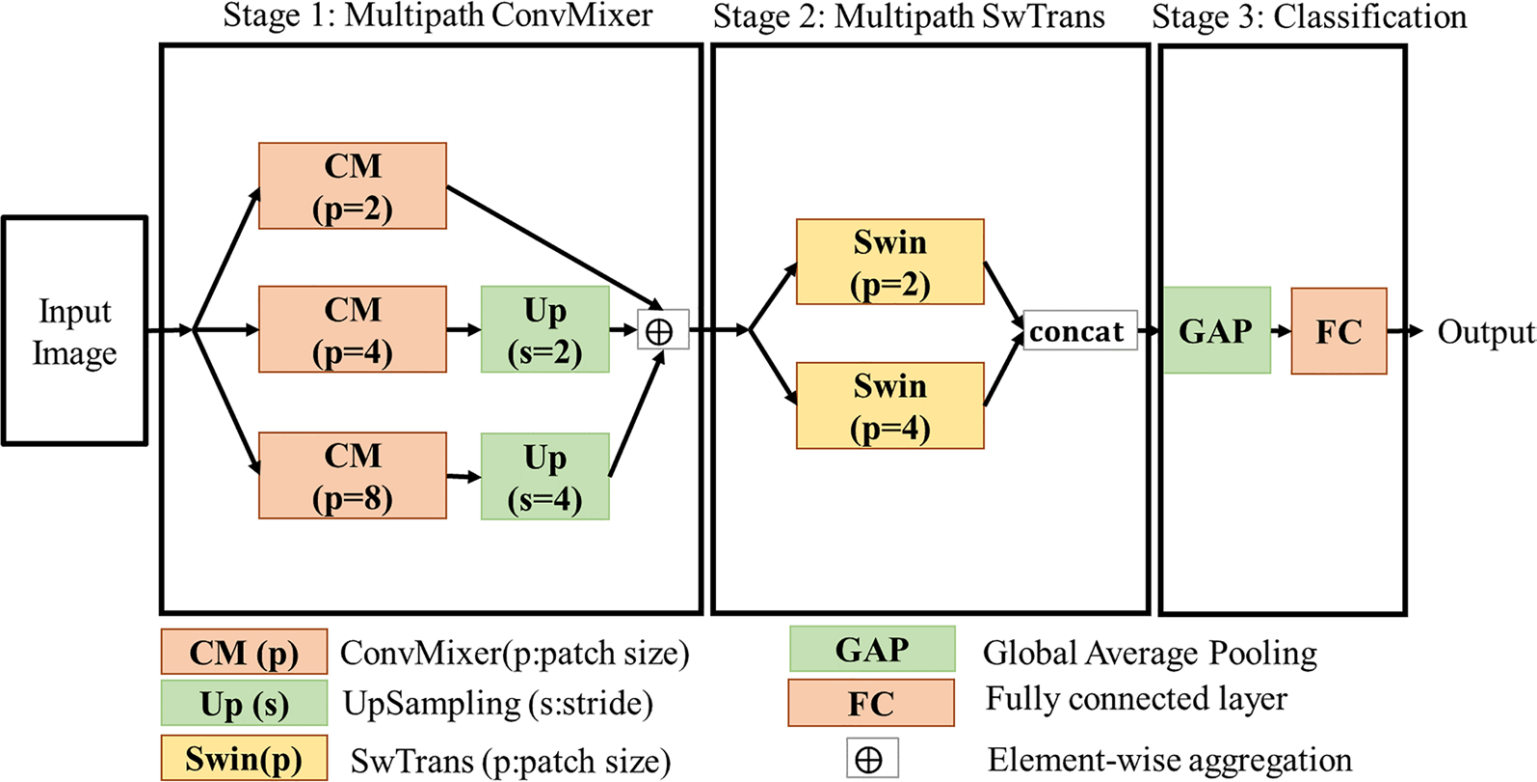
Proposed method

In this study, a new hybrid method based on the popular ConvMixer and SwTrans is proposed. This model, called SwTrans and ConvMixer based Multipath mixer (SC-MP-Mixer), is given in Fig. 2.

In this section, the SC-MP-Mixer module is discussed in three stages. In the first stage, three different ConvMixers were applied parallel to the input image. The main idea here is to extend the notion of patches that the ConvMixer model is based on. Starting from this point, three different patch sizes were used while obtaining the patches. Then ConvMixer was applied for each patch and finally ConvMixer combined the outputs. In the combining process, element-based aggregation was performed.

In the second stage of the SC-MP-Mixer model, powerful long-range contextual information is obtained by using ConvMixer outputs. At this point, the Swin transformer model, which is more economical than the ViT model, is used. In addition, as in the first stage, a Swin transformer was applied in two different ways, with patch sizes of 2 and 4. The two outputs obtained at the end of the second stage were combined. The final stage of the SC-MP-Mixer is the classification stage. GAP (Global Average Pooling) fully connected layer was applied to the feature map obtained at the end of the second stage. Finally, the classification prediction output is obtained by applying the softmax layer to the fully connected layer output.

In the rest of this section, the stages of the SC-MP-Mixer are discussed in detail.

#### Stage 1: multipath ConvMixer (MPCM)

**Fig. 3:**
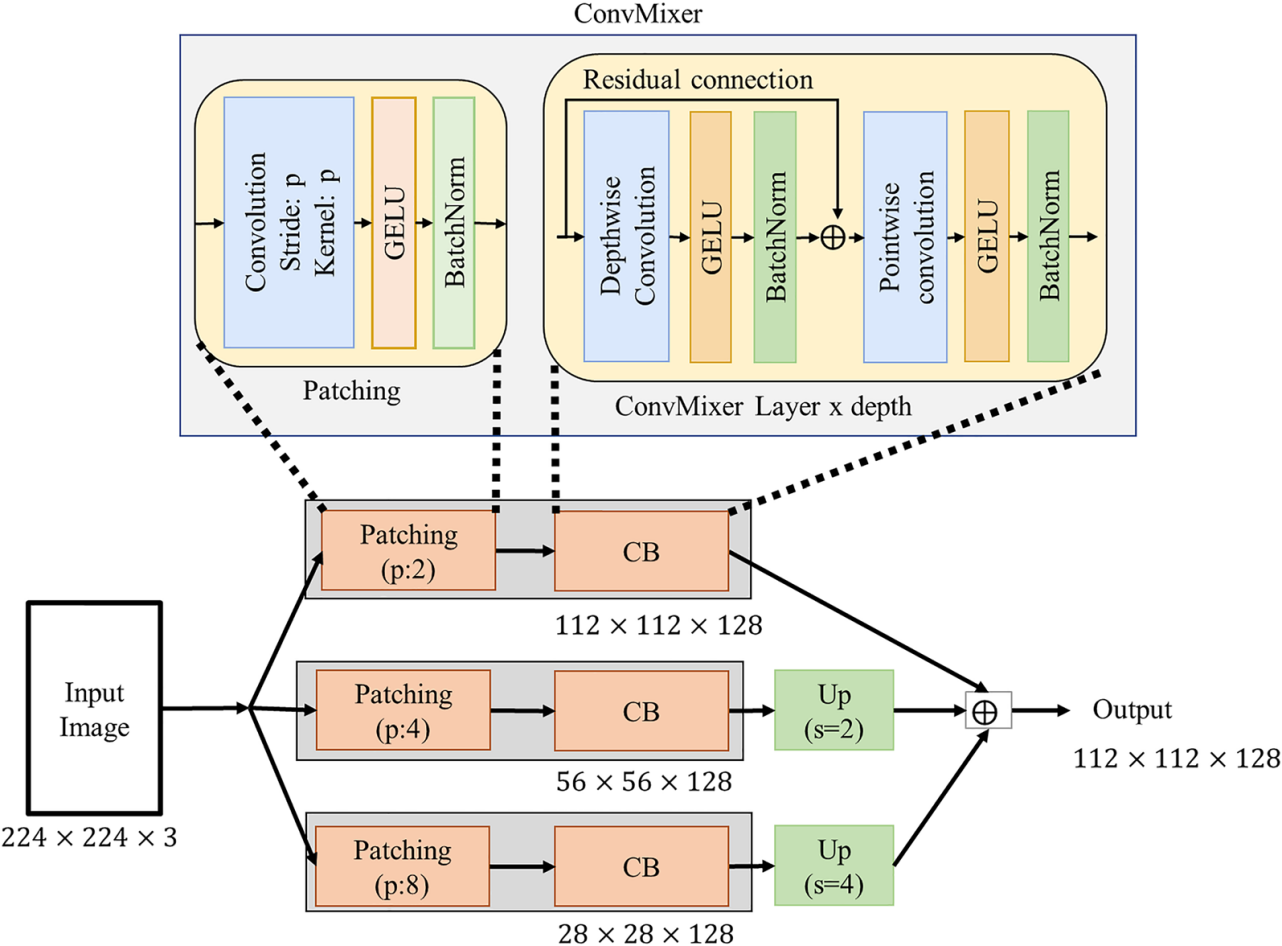
Multipath ConvMixer (MPCM)

The main starting point of the ConvMixer model is to obtain an affordable and high-performance model by treating the image in patches. However, the selection of an effective patch size poses a new problem. Some images, especially images of WBCs, often have a very similar background. In addition, the object size of the objects in the images is uncertain and homogeneous. Based on this problem, the Multipath ConvMixer (MPCM) model was developed for the effective patch width for ConvMixer in the SC-MP-Mixer model. The proposed structure is shown in Fig. 3.

As can be seen in Fig. 3, 3 ConvMixer blocks are used in parallel in the MPCM model. In the patching block, which is the first stage of these ConvMixer blocks, the image is divided into patches. The patch size (p) value used in this block is 2, 4 and 8, respectively. Then the patching output fed the ConvMixer layers. Depthwise convolution and pointwise convolution operations were performed in ConvMixer blocks, respectively. In addition, GELU activation and BN layer were applied after each convolution operation. There are also resudial connections in the ConvMixer block as shown in Fig. 3. In the MPCM model, the depth value of each ConvMixer block is taken as 4. In addition, the kernel size of the Depthwise convolution is 3 and the filter number of the pointwise convolution is 128 in ConvMixer blocks.

Toward the output of the MPCM model, each ConvMixer outputs feature maps are combined with element-wise aggregation operation. For the merging process, the feature maps of three different sizes were first brought to the same size as the UpSampling layer. Then, element-wise aggregation process was applied. This process is formulated as follows:

4 $$F_{output} = F_{C1} \oplus Up^{s = 2} \left( {F_{C2} } \right) \oplus Up^{s = 4} \left( {F_{C3} } \right)$$

In Eq. (4), $F_{C1}$, $F_{C2}$ and $F_{C3}$ are the output feature maps of the ConvMixer blocks shown in Fig. 3. On the other hand, $Up^{s}$ represents the UpSampling layer with $s$ stride value. Finally, $F_{output}$ is the feature map acquired in the output of the MPCM module.

#### Stage 2: multipath Swin transformer (multipath SwTrans-MPST)

The ConvMixer model has been applied in many studies and its superiority has been proven. On the other hand, SwTrans models with self-attention mechanisms are very effective in obtaining long-range context information. Therefore, in the SC-MP-Mixer model, SwTrans blocks are used to obtain long-range contextual information models.

**Fig. 4:**
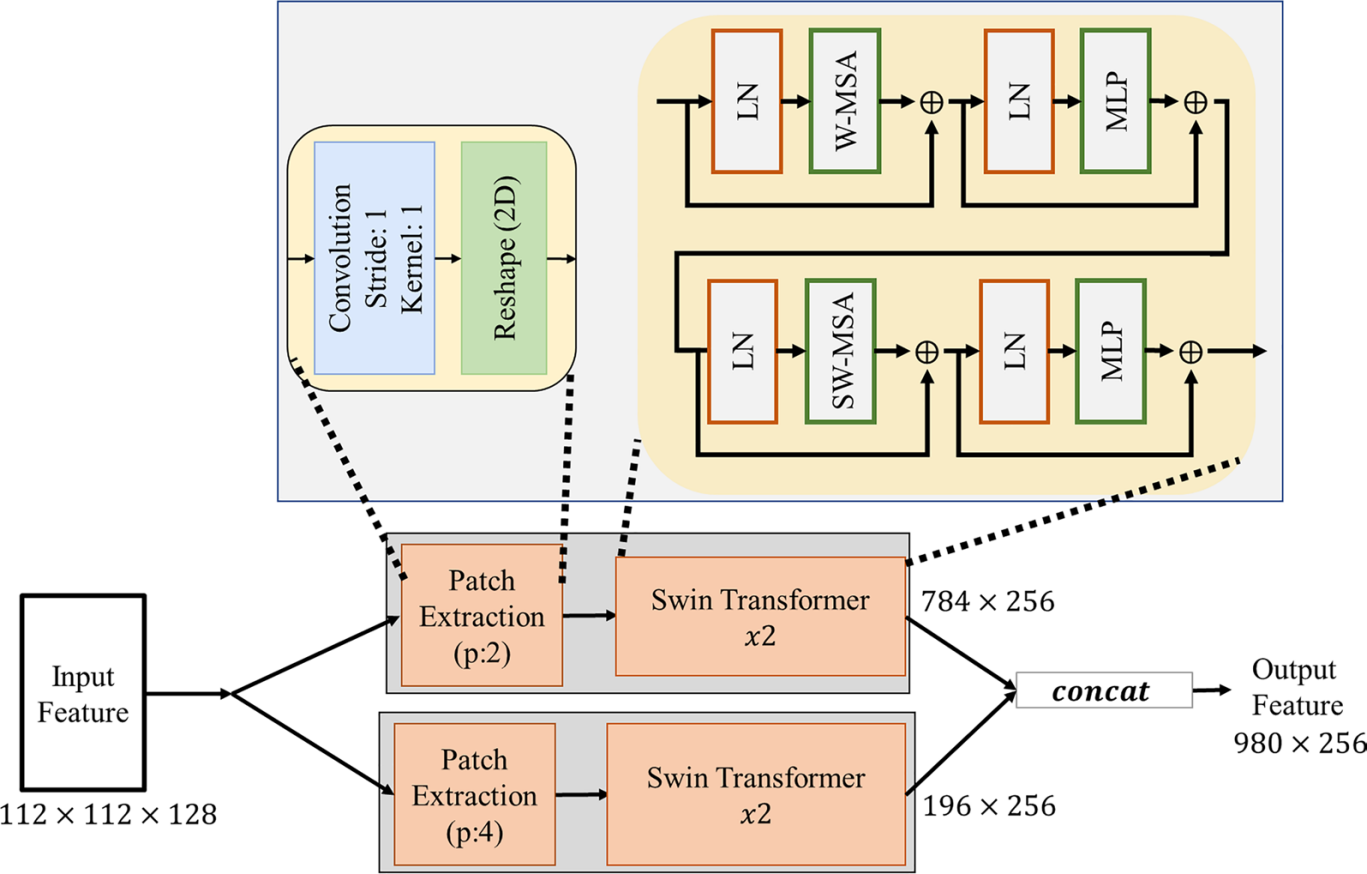
Multipath SwTrans (MPST)

In the second stage of the proposed SC-MP-Mixer model, two SwTrans blocks with different patch sizes are used as in the MPCM model. Thanks to this structure called Multipath SwTrans (MPST) module, it has been observed that stronger global semantic features are obtained (see experimental study). The proposed MPST model is shown in Fig. 4.

As shown in Fig. 4, two different SwTrans blocks are applied in parallel in the MPST model. SwTrans blocks are based on handling the relationship between patches using the self-attention mechanism. Therefore, the input image has to be split into patches. In the Patch Extraction process suggested in the MPST blog, a convolution process is applied first. The applied convolution operation is formulated as in Eq. (5).

5 $$L_{112 \times 112 \times k} = Conv^{k} \left( {F_{112 \times 112 \times 128} } \right)$$

The $k$ shown in Eq. (5) represents the number of filters. The $k$ value is determined by the formula $k = 256/p^{2}$ ($p$ is patch size). The main purpose of this formula is to avoid excessive processing cost caused by self-attention applied in SwTrans. In the second step of the Patch Extraction process, the $112 \times 112 \times k$ feature map is divided into patches of $p \times p \times k$ size. After fragmentation, $112/p \times 112/p$ patch was obtained. Finally, each patch obtained was vectorized and combined. As a result of the process, a vectorized feature map of $112/p \, 112/p \times p^{2} k$ was obtained. Since $k$ value is given as $k = 256/p^{2}$, the $p^{2} k$ result given here is obtained as 256. These operations are shown as in Eq. (6).

6 $$M_{{\frac{112}{p}.\frac{112}{p} \times 256}} = Reshape\left( {patching\left( {L_{112 \times 112 \times k} } \right)} \right)$$

The M feature map shown in Eq. (6) represents the feature map from which the swin transforms are fed. In the MPST model, SwTrans operation was applied to the M feature map twice. Details of the SwTrans are given in Sect. “Swin transformer (SwTrans)”. As shown in Fig. 4, the patch size value in the patch extraction process applied in parallel in the MPST model is taken as 2 and 4, respectively. On the other hand, the number of neurons of the MLP in the SwTrans is 256.

#### Stage 3: Classification

The final stage of the proposed SC-MP-Mixer model is the classification stage. At this stage, the features obtained from the SwTranss were first combined. As shown in Fig. 4, a 980 × 256 feature map was obtained as a result of the concatenating process. Then, the GAP process was implemented to the obtained feature map. Finally, the FC and softmax layer are used to obtain a classification prediction output. These processes are expressed as follows.

7 $$\begin{gathered} F_{final} = GAP\left( {concat\left( {N_{784 \times 256} ,N_{196 \times 256} } \right)} \right) \hfill \\ prediction = Softmax\left( {FC_{c} \left( {F_{final} } \right)} \right) \hfill \\ \end{gathered}$$

Shown here is the final feature map used for the $F_{final}$ classification, measuring 980 × 256. It is a FC layer with $c$ neurons used for $FC_{c}$ classification. Finally, the value of $c$ represents the number of classes.

The categorical Cross-Entropy loss function was used in training the proposed SC-MP-Mixer architecture. The formalization of the Categorical Cross Entropy loss function is shown in Eq. (8).

8 $$L = - \mathop \sum \limits_{{\text{k}}}^{{\text{M}}} {\text{Y}}_{{\text{k}}} {\text{log}}\left( {{\text{P}}_{k} } \right)$$

where L denotes the loss value of the classification network. Y and P denote the expected and prediction, respectively. M represents the number of classes and *k* represents the index of classes.

### Blood cell datasets

**Fig. 5:**
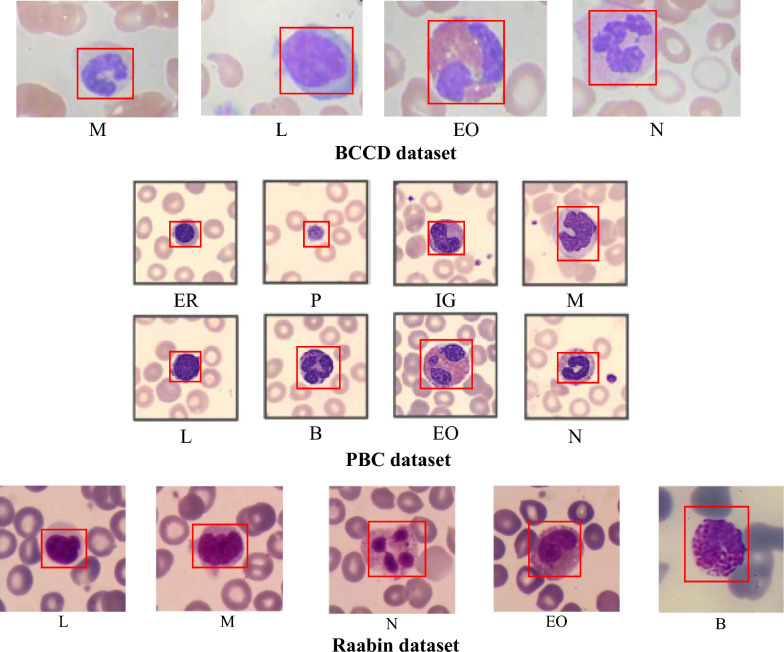
Representative images for WBC subtypes included in the Raabin, PBC, and BCCD datasets

To evaluate the classification outcomes of our SC-MP-Mixer model in this study, experiments were conducted using three distinct WBC image datasets. The initial dataset, BCCD, encompasses four WBC types: Neutrophils (N), Eosinophils (EO),Lymphocytes (L) and Monocytes (M). It comprises a total of 12,444 microscopic WBC images, each sized at 320 × 240 pixels and formatted as RGB images [44]. Within BCCD dataset, individual folders include training and testing images corresponding to each of the four WBC types. The BCCD encompasses a total of 12,444 microscopic WBC images, comprising 2499 N, 2478 M, 2483 L, and 2497 EO images for training, along with 623 EO, 620 L, 620 M and 624 N images for testing. The second dataset (PBC) originates from Anna et al. and is publicly accessible, gathered at the Barcelona hospital clinic [3]. The PBC dataset encompasses a total of 17,092 PBC images. These images were captured from healthy individuals without any pharmacological treatment, infectious diseases, hematological or oncological. The images in the PBC dataset are in RGB format, sized at 360 × 363 pixels. Moreover, specialized pathologists at the hospital provided labels for these images. The dataset comprises 8 distinct WBC types, namely N, EO, L, M, Basophils (B), Platelets (P), immature granulocytes (IG) such as promyelocytes, myelocytes, metamyelocytes and Erythroblasts (ER). The Raabin dataset, a sizable open-access collection released in 2021, constitutes the third dataset analyzed in this study [45]. Within this dataset, three distinct sets of cropped WBC images exist: Train, Test-A, and Test-B. While both the Train and Test-A sets underwent labeling by two separate experts, the Test-B images lack comprehensive labeling. Consequently, our study focused solely on the Train and Test-A sets, sourced from 56 ordinary peripheral blood smears (representing L, M, N, and EO) and one instance of chronic myeloid leukemia (representing basophil). The Raabin dataset encompasses five WBC types (M, L, EO, N, and B), totaling 14,514 microscopic WBC images. Among these are 212 B, 6231 N, 561 M, 2427 L and 744 EO images for training, and 89 B, 322 EO, 1034 L, 234 M and 2660 N images for testing purposes. Table 1 contains details about WBC types and sample images across three datasets. Furthermore, Fig. 5 showcases illustrative images representing WBC subtypes present in the Raabin, PBC, and BCCD datasets.

**Table 1:** Detailed information about the BCCD, PBC and Raabin datasets

Dataset	Blood cell types	ER	P	IG	M	L	B	EO	N	Total
BCCD	Number of images	–	–	–	3098	3103	–	3120	3123	12,444
PBC	Number of images	1551	2348	2895	1420	1214	1218	3117	3329	17,092
Raabin	Number of images	–	–	–	795	3461	301	1066	8891	14,514

## Experimental studies and results

Numerous experimental investigations were conducted to meticulously analyze the classification accuracy of our SC-MP-Mixer model. This section presents these experimental studies. Within this section’s progression, initial focus is placed on elaborating the parameter settings. Subsequently, the classification performance outcomes obtained from experiments conducted on three datasets utilizing our SC-MP-Mixer model are delineated. Lastly, comparisons were drawn with analogous studies found in the existing literature.

### Parameter settings

The experimental studies utilized BCCD, PBC and Raabin datasets. The BCCD dataset was originally allocated for 9957 samples in training and 2487 samples in testing. To ensure a fair comparison, we used the training and test examples in the dataset as is. Additionally, 15% of the training samples were set aside for validation purposes. In contrast, for the PBC and Raabin datasets, the training and test samples were not separated in the original dataset, so we partitioned them into specific proportions. The PBC and Raabin dataset was divided into 70% training, 15% validation, and 15% testing.

The experimental studies were conducted on a computer equipped with an Intel i9 processor, 64 GB RAM, and an RTX 3080 Ti graphics card. The design of the deep learning model utilized the Python programming language and the Keras-TensorFlow library. In the training of deep learning models, a learning rate of 0.0001, a batch size of 16, and 100 epochs were employed. Additionally, the Adam method was used for parameter optimization.

In the first stage of the proposed SC-MP-Mixer module, 3 different ConvMixers (CM1, CM2, and CM3) were used in parallel with the input image. The p (patch size) values were 2, 4, and 8 for CM1, CM2, and CM3, respectively. In addition, the number of filters and depth size used in all ConvMixer models were 128 and 4, respectively. Additionally, the s values used in the upsampling layers were set to 2 and 4 (see “Proposed method” or “Stage 1: Multipath ConvMixer (MPCM)” Sects.). On the other hand, two Swin Transformers were used in the second part of the proposed model. The p values here are set to 2 and 4, respectively, for the patching process of each Swin Transformer (see “Proposed method” or “Stage 2: multipath Swin transformer (multipath SwTrans-MPST)” Sects.). Finally, the c values in the classification process were set as 4 for the BCCD dataset, 8 for the PBC dataset, and 5 for the Raabin dataset.

The evaluation of the SC-MP-Mixer model’s efficiency was performed using criteria such as classification accuracy (Acc), recall (Re), F1-score (F1s) and precision (Pr). These evaluation criteria provide an objective and quantitative measure of the model’s prediction effectiveness, essential for performance assessment and identifying areas for improvement. The formulas of these metrics is given in Eqs. (9–12).

9 $$Acc = \frac{TP + TN}{{TP + FP + TN + FN}}$$

10 $$Pr = \frac{TP}{{TP + FP}}$$

11 $$Re = \frac{TP}{{TP + FN}}$$

12 $$F1s = 2*\frac{Pr* Re}{{Pr + Re}}$$

The Eqs. (9–12) derive the true positives (TP), false positives (FP), false negatives (FN) and true negatives (TN) values from the confusion matrix. They represent: FP as the number of WBC inaccurately identified as non-target WBC types, FN as the total of incorrectly identified WBC types, TN as the tally of WBC correctly recognized as non-target WBC types and TP as the count of correctly identified WBC types.

### Experimental results

In this section, the results of the applications on the BCCD, PBC and Raabin datasets are included. These three datasets were compared with ResNet (ResNet50, ResNet101) [46], EfficientNet [47], ConvMixer [22], Swin Transformer [14], MobileNet [48] and VGG16 [49] from the literature. The reasons for selecting these methods for comparison are as follows. The proposed model uses parallel ConvMixer and Swin Transformer models. In order to observe the effect of using only ConvMixer and Swin Transformer, the proposed model is first compared with these two models. Certain models, including EfficientNet, MobileNet, ResNet101, ResNet50, and VGG16, were chosen for comparison in the experiments due to their widespread adoption and established performance in the computer vision field. Each of these models represents a different architectural paradigm offering varying balances between model complexity, computational efficiency, and accuracy. The VGG16 is a classical deep convolutional neural network architecture characterized by its simplicity and uniform architecture. It is widely used as a baseline for many computer vision tasks due to its effectiveness and ease of implementation. The ResNet50 and ResNet101 architectures pioneered the use of skip connections and achieved superior performance by developing very deep network structures. Finally, the MobileNet and EfficientNet architectures are efficient network architectures with low parameters that utilize both skip connections and depthwise separable convolution layers. Additionally, the EfficientNet architecture offers a powerful network structure by performing layer optimization. These network architectures are actively used in many studies today, either in their original or modified versions. In addition, they also include skip connections and depthwise separable convolution layers, as in our proposed model. Discussions of the results are provided below in subsections.

#### Classification results for BCCD

The BCCD consists of four classes: N, EO, L and M. Class-based results for BCCD are presented in Table 2. Table 2 shows that the best results are obtained for class L with 99.8% Acc, 99.60% F1s, 99.68% Pr and 99.52% Re. This shows that the model performs extremely well in identifying L class cells, achieving very high accuracy and other performance metrics. It also shows that the model is very good at both identifying true positives and avoiding false positives, with the model rarely making errors for class L. The proposed model performs well on class M with high Acc (98.47%) and F1s (96.84%). Notably, it has a perfect Pr (100%), meaning it never classified anything else as M if it wasn’t actually M. However, the Re (93.87%) is slightly lower, suggesting there might be some true positives from class M that the model missed. The proposed model exhibits good Acc (92.88%) for the EO class, however, improvements are needed in its F1s (85.53%), Re (83.95%), and Pr (87.17%). A slightly higher Pr compared to Re suggests that the model might be better at avoiding false positives (misclassifying other classes as EO), but it could potentially miss some true positives (actual EO data points). Class N has the lowest performance among the other classes. The Acc (91.48%) is still good, but the F1s (83.82%) is the lowest. The Pr (80.03%) is also lower than the Re (87.98%), indicating that the model may struggle to distinguish class N from other classes. Overall, the proposed model performs well on the BCCD dataset, especially on class L. However, improvements could be made in distinguishing classes EO and N from other classes, especially in Pr for class N and Re for class EO. In additon, Table 3 outlines the application results of different models using this dataset. Upon examination of Table 3, it is apparent that the proposed SC-MP-Mixer achieved the best classification result, boasting an macro Acc of 95.66%. Additional evaluation criteria for our SC-MP-Mixer method yielded the following results: 91.44% F1s, 91.72% Pr, and 91.33% Re. Comparatively, the results closest to the our SC-MP-Mixer model was acquired with EfficientNet, achieving 93.87% Acc and 88.05% F1s. Similarly, ResNet101 attained 93.65% Acc and 87.61% F1s. The ResNet50 reached Acc score of 93.14%, while the MobileNet achieved Acc score of 92.22%. The VGG16 obtained the lowest scores among pre-trained models. When comparing pre-trained models with the SC-MP-Mixer, it produced approximately 3–5% higher scores than the pre-trained models. These results demonstrate the superior effectiveness of the SC-MP-Mixer model over pre-trained models. On the other hand, the Swin Transformer and ConvMixer structures, representing the latest technological models, achieved Acc scores of 92.62 and 93.18%, respectively. Despite the Swin Transformer capturing strong long-context features with the latest transformer technology, it exhibited weaknesses against the proposed model. This is attributed to transformer models struggling to capture spatial details from input images and requiring a large number of images for training. In the SC-MP-Mixer architecture developed based on this, Swin Transformers were fed with features obtained from ConvMixer, allowing for numerous and effective features through a multi-path approach. Consequently, the proposed SC-MP-Mixer produced the highest results by leveraging the Swin Transformer and ConvMixer blocks together.

**Table 2:** Class-based results for BCCD (%)

Classes	Acc	F1s	Pr	Re
EO	92.88	85.53	87.17	83.95
L	99.8	99.60	99.68	99.52
M	98.47	96.84	100	93.87
N	91.48	83.82	80.03	87.98
Macro results	95.66	91.44	91.72	91.33

**Table 3:** Classification results of different models for BCCD (%)

Model	Macro Acc	F1s	Pr	Re
ConvMixer	93.18	87.03	90.91	86.36
EfficientNet	93.87	88.05	90.56	87.73
MobileNet	92.22	84.52	88.06	84.43
ResNet101	93.65	87.61	89.99	87.29
ResNet50	93.14	86.7	89.73	86.29
SwinTransformer	92.62	85.36	85.72	85.25
VGG16	62.53	10.02	6.26	25.05
**Proposed** **SC-MP-Mixer**	**95.66**	**91.44**	**91.72**	**91.33**

Bold indicates the best result

#### Classification results for PBC and Raabin

The PBC dataset comprises eight classes: N, EO, B, L, M, IG, P and ER. Class-based results for PBC are presented in Table 4. Analysing Table 4, we can see that the proposed method generally performs well in all classes, with some differences. Classes B, EO, L, ER and P achieved very high Acc (over 99.8%) and F1s (over 99%). They also have Pr and Re values close to 100%, indicating that the model excels in classifying these classes with minimal error. Classes M and N show good Acc (over 98.9%) but slightly lower F1s (around 97–99%). The Pr and Re values for these classes are still quite high, suggesting that the model performs well but may have some room for improvement in distinguishing these classes from others. Class IG has the lowest Acc (98.91%) and F1s (96.70%) of all classes. In addition, the Pr (96.93%) is slightly higher than the Re (96.48%), indicating that the model may be better at avoiding false positives for this class, but may miss some true positives. Overall, the model performs well on the PBC dataset, particularly in the B, EO, L, ER and P classes. Some improvement is needed in the IG class, particularly in Re, and potentially in the M and N classes. The application results using this dataset are summarized in Table 5. According to the Table 5, the most notable classification outcomes were obtained with our proposed SC-MP-Mixer. The SC-MP-Mixer demonstrated impressive performance, achieving 99.65% Acc, 98.71% F1s, 98.67% Pr, and 98.76% Re values. In comparison, the methods that closely approached the performance of the proposed SC-MP-Mixer on this dataset were ResNet50 and VGG16. ResNet50 achieved a 99.53% Acc score, while VGG16 obtained a 99.59% Acc score. Additionally, the EfficientNet, MobileNet, and ResNet101 models provided Acc scores of 99.51, 99.04, and 99.35%, respectively. On the other hand, ConvMixer (98.51%) and SwinTransformer (97.9%) yielded the lowest Acc scores. However, the SC-MP-Mixer, utilizing both ConvMixer and SwinTransformer in tandem and supported by a multi-path (parallel) application and a different patch size approach, outperformed ConvMixer and SwinTransformer by 1.14 and 1.75%, respectively.

**Table 4:** Class-based results for PBC (%)

Classes	Acc	F1s	Pr	Re
B	99.96	99.71	99.42	100
EO	99.96	99.90	100	99.80
ER	99.84	99.10	99.10	99.10
IG	98.91	96.70	96.93	96.48
L	99.88	99.14	100	98.29
M	99.61	97.68	96.33	99.06
N	99.02	97.45	97.55	97.35
P	100	100	100	100
Macro results	99.65	98.71	98.67	98.76

**Table 5:** Classification results of different models for PBC (%)

Model	Macro Acc	F1s	Pr	Re
ConvMixer	98.51	93.26	93.6	93.53
EfficientNet	99.51	98.02	97.88	98.25
MobileNet	99.04	96.07	96.25	95.98
ResNet101	99.35	97.08	97.25	96.97
ResNet50	99.53	98.29	98.25	98.33
SwinTransformer	97.9	90.35	91.78	90.75
VGG16	99.59	98.33	98.39	98.28
**Proposed** **SC-MP-Mixer**	**99.65**	**98.71**	**98.67**	**98.76**

Bold indicates the best result

The Raabin dataset comprises five classes: N, EO, B, L and M. Class-based results for Raabin are presented in Table 6. When analysing Table 6, the performance of the model varies according to the classes in this dataset. Class N achieves the highest F1s (98.55%) with very close Pr (98.40%) and Re (98.70%). This suggests that the model is good at identifying and correctly classifying class N with minimal error. Compared to class N, classes B and EO have good accuracy (over 99%) but lower F1 values (around 94–98%). While the precision for class B is excellent (100%), the recall is lower (96.30%), indicating that the model may miss some true positives (B) but is successful in avoiding false positives. Similarly, Pr and Re are lower in the EO class. Class L has the lowest Acc value, while class M has the lowest Pr, Re and F1s values. This indicates that the model has difficulty distinguishing class M from the others and may make more errors in its classifications. Overall, the model seems to give a balanced result of class N in all evaluation metrics in the Raabin dataset. Although the highest Acc value was obtained in class B, it gave lower results than class N, especially in F1s and Re. It is clear that the model needs improvement, especially for class M. In addition, The experimental results using this dataset are summarized in Table 7. According to the Table 7, the most notable classification outcomes were obtained with our proposed SC-MP-Mixer. The SC-MP-Mixer demonstrated impressive performance, achieving 98.68% Acc, 94.42% F1s, 94.34% Pr, and 94.63% Re values. The closest result to the proposed SC-MP-Mixer was achieved in MobileNet with 97.71% Acc, 93.86% F1s, 93.79% Pr, and a 93.96% Re. When compared to MobileNet, the proposed SC-MP-Mixer seems to yield superior results by 0.97% in Acc, 0.56% in F1s, 0.55% in Pr, and 0.67% in Re. Furthermore, when pitted against ConvMixer, the suggested SC-MP-Mixer attains notably improved outcomes with an increase of 21.33% in Acc, 37.95% in F1s, 27.56% in Pr, and 38.14% in Re. Likewise, in comparison to the SwTrans, the SC-MP-Mixer achieves superior results with a marginal uptick of 2.13% in Acc, 0.8% in F1s, 0.82% in Pr, and 0.91% in Re. Against other methodologies, the SC-MP-Mixer surpasses EfficientNet by 31.25% in Acc, 44.34% in F1s, 45.06% in Pr, and 37.68% in Re. Correspondingly, it outperforms ResNet101 with a slight increase of 2.12% in Acc, 2.34% in F1s, 3.71% in Pr, and 1.05% in Re, surpasses ResNet50 with 2.17% higher Acc, 2.87% higher F1s, 4.97% higher Pr, and 0.79% higher Re, and outshines VGG16 with a significant lead of 37.08% in Acc, 69.05% in F1s, 64.55% in Pr, and 58.58% in Re.

**Table 6:** Class-based results for Raabin (%)

Classes	Acc	F1s	Pr	Re
B	99.91	98.12	100	96.30
EO	99.27	94.84	96.08	93.63
L	97.75	95.31	96.51	94.14
M	98.21	85.28	80.71	90.4
N	98.25	98.55	98.40	98.70
Macro results	98.68	94.42	94.34	94.63

**Table 7:** Classification results of different models for Raabin (%)

Model	Macro Acc	F1s	Pr	Re
ConvMixer	77.35	56.47	66.78	56.49
EfficientNet	67.43	50.08	49.28	56.95
MobileNet	97.71	93.86	93.79	93.96
ResNet101	96.56	92.08	90.63	93.58
ResNet50	96.51	91.55	89.37	93.84
SwinTransformer	96.55	93.62	93.52	93.72
VGG16	61.60	25.37	29.79	36.05
**Proposed** **SC-MP-Mixer**	**98.68**	**94.42**	**94.34**	**94.63**

Bold indicates the best result

**Fig. 6:**
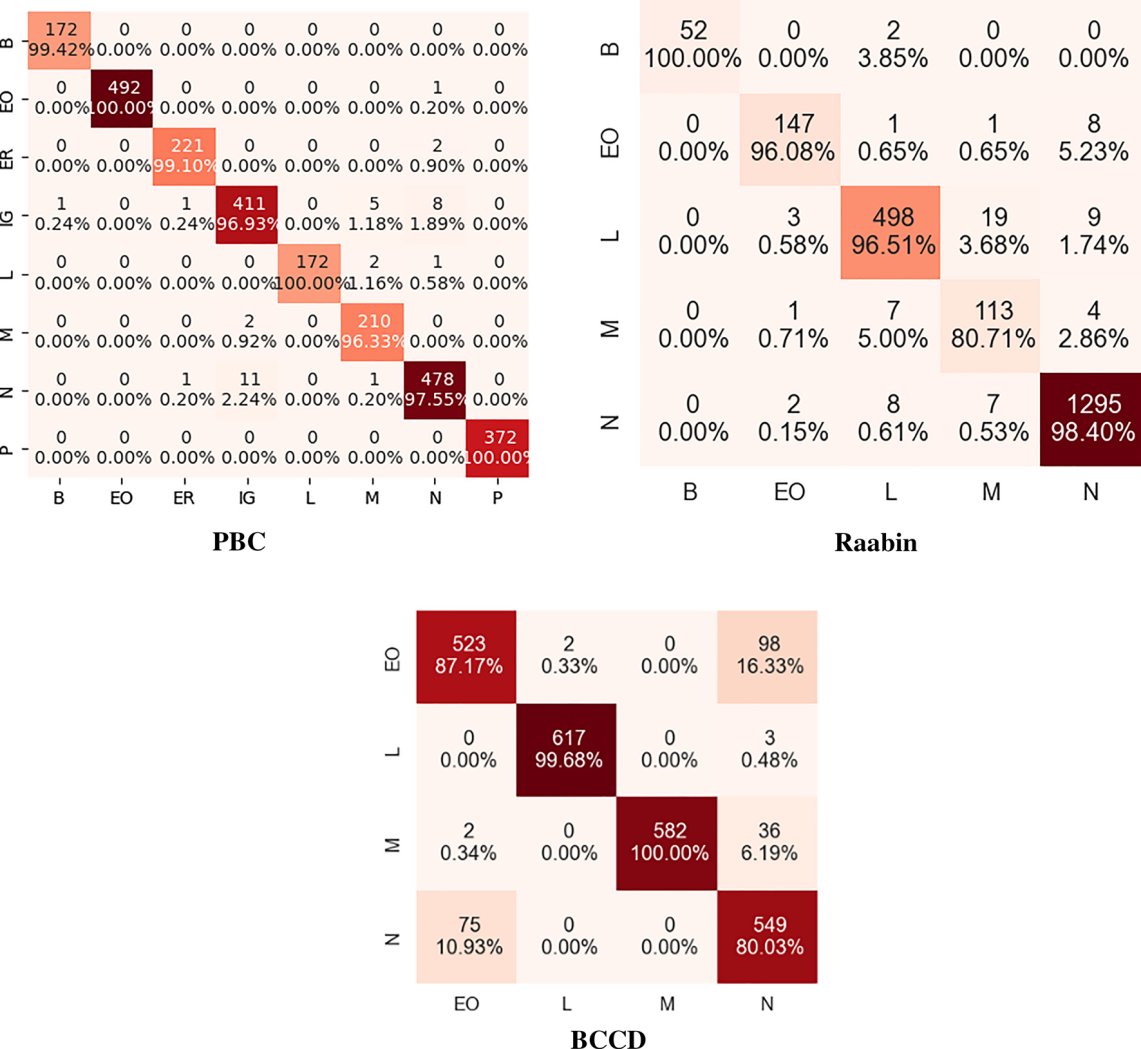
Confusion matrices obtained for each data set using the proposed SC-MP-Mixer. The *x*-axis and *y*-axis of the complexity matrices indicate the predicted label and the true label, respectively

The confusion matrices obtained from the experimental studies conducted using the proposed SC-MP-Mixer with all three datasets are presented in Fig. 6. According to Fig. 6, the proposed SC-MP-Mixer correctly predicted all 172 B images, 492 out of 493 EO images, 221 out of 223 ER images, 411 out of 426 IG images, 172 out of 175 L images, 210 out of 212 M images, 478 out of 491 N images, and all 372 P images in the PBC dataset. Similarly, in the Raabin dataset, it correctly predicted 52 out of 54 B images, 147 out of 157 EO images, 498 out of 529 L images, 113 out of 125 M images, and 1295 out of 1312 N images. Finally, within the BCCD dataset consisting of 4 classes, it correctly predicted 523 out of 623 EO images, 617 out of 620 L images, 582 out of 620 M images, and 549 out of 624 N images. Considering the total correctly predicted images, class-specific accuracy values for each dataset are provided in Table 2 for BCCD, Table 4 for PBC, and Table 6 for Raabin. The macro Acc values obtained using the proposed SC-MP-Mixer for BCCD, PBC, and Raabin datasets are as follows: 95.66, 99.65, and 98.68%, respectively.

#### Ablation analysis

The proposed SC-MP-Mixer model comprises a combination of Multipath SwTrans (MPST) and Multipath ConvMixer (MPCM) architectures. While the MPCM structure consists of three parallel ConvMixer blocks, the MPST structure comprises two parallel SwTrans blocks. The individual impact of each component within the proposed SC-MP-Mixer model on classification results (Acc, Pr, Re, and F1s) has been examined and presented in Table 8. Model 1 contains only ConvMixer, Model 2 includes solely SwTrans, Model 3 incorporates solely the MPCM structure, Model 4 integrates both MPCM and SwTrans, Model 5 contains only MPST, Model 6 is the combination of ConvMixer and MPST, and finally, Model 7 encompasses the components within the proposed SC-MP-Mixer model.

**Table 8:** Results of ablation analysis

Model	ConvMixer	MPCM	SwTrans	MPST	BCCD (%)	PBC (%)	Raabin (%)
Model 1	X	–	–	–	Acc = 93.18	Acc = 98.51	Acc = 77.35
					Pr = 90.91	Pr = 93.6	Pr = 66.78
					Re = 86.36	Re = 93.53	Re = 56.49
					F1s = 87.03	F1s = 93.26	F1s = 56.47
Model 2	–	–	X	–	Acc = 92.62	Acc = 97.9	Acc = 96.55
					Pr = 85.72	Pr = 91.78	Pr = 93.52
					Re = 85.25	Re = 90.75	Re = 93.72
					F1s = 85.36	F1s = 90.35	F1s = 93.62
Model 3	–	X	–	–	Acc = 93.24	Acc = 98.77	Acc = 97.34
					Pr = 90.14	Pr = 97.74	Pr = 92.25
					Re = 89.48	Re = 97.59	Re = 92.46
					F1s = 89.81	F1s = 97.66	F1s = 92.35
Model 4	–	X	X		Acc = 94.39	Acc = 99.54	Acc = 98.44
					Pr = 90.43	Pr = 98.48	Pr = 92.88
					Re = 89.99	Re = 98.19	Re = 93.14
					F1s = 90.21	F1s = 98.33	F1s = 93.01
Model 5	–	–	–	X	Acc = 93.12	Acc = 98.64	Acc = 97.18
					Pr = 89.20	Pr = 97.87	Pr = 91.47
					Re = 88.79	Re = 97.28	Re = 91.65
					F1s = 88.99	F1s = 97.57	F1s = 91.56
Model 6	X	–	–	X	Acc = 94.08	Acc = 99.04	Acc = 98.01
					Pr = 90.12	Pr = 98.11	Pr = 91.96
					Re = 89.65	Re = 97.99	Re = 91.37
					F1s = 89.88	F1s = 98.05	F1s = 91.66
Model 7	–	X	–	X	Acc = 95.66	Acc = 99.65	Acc = 98.68
					Pr = 91.72	Pr = 98.67	Pr = 94.34
					Re = 91.33	Re = 98.76	Re = 94.63
					F1s = 91.44	F1s = 98.71	F1s = 94.42

Analysing Table 8, the lowest classification results are obtained when the ConvMixer (Model 1) and SwTrans (Model 2) models are used alone. When comparing the results between Model 1 and Model 3 in Table 8, Model 3 has yielded a respective improvement of 0.06%, 0.26%, and 19.99% in accuracy for the BCCD, PBC, and Raabin datasets compared to Model 1. These outcomes indicate that the MPCM structure is more effective than a single ConvMixer block. MPST (Model 5), when used alone, achieves lower accuracy compared to models with other components except for ConvMixer only and SwTrans only. However, the inclusion of MPST with ConvMixer (Models 6 and 7) improves performance for BCCD, PBC and Raabin. This suggests that MPST, when combined with ConvMixer, may be helpful in addressing certain aspects of the data. Furthermore, including ConvMixer consistently improves performance across all datasets. This suggests that ConvMixer effectively extracts important features from the images. Moreover, in Model 4, augmenting the SwTrans model to the MPCM model exhibits further enhancement: the accuracy values increase by 1.15, 0.77, and 1.1% for the BCCD, PBC, and Raabin datasets, respectively. Introducing the MPST block instead of a single SwTrans model alongside the MPCM model leads to an increase in accuracy by 1.27, 0.11, and 0.24% for the BCCD, PBC, and Raabin datasets, respectively. When all models are analyzed, it is seen that Model 7, the proposed SC-MP-Mixer model, achieves the most successful results in all evaluation metrics in all datasets.

#### Comparison analysis with different models in the literature

To demonstrate the effectiveness of our SC-MP-Mixer model, we compared it with different studies from the literature. The comparison results are shown in Table 9. In these comparisons, our proposed model was repeated 4 times, and its standard deviation was calculated and added to this table. When Table 9 is examined, it is seen that the SC-MP-Mixer method achieved the best results in BCCD, PBC and Raabin datasets. In the BCCD dataset, our SC-MP-Mixer method achieved 95.66% macro Acc. In this dataset, the closest result to the SC-MP-Mixer method was found with the proposed Canonical Correlation Analysis (CCA)—(InceptionV3 + LSTM) method by Patil et al. [1] with 91.06%. The SC-MP-Mixer method gives 4.6% better results. In addition, Patil et al. [1] found an Acc of 89.85% when using VGG16 in conjunction with LSTM. The SC-MP-Mixer method achieved a 5.81% higher Acc value than VGG16 + LSTM. Similarly, our SC-MP-Mixer method has 4.65% better Acc than Bani-Hani et al. [24] method (CNN + Genetic Algorithm) and 4.87% better than Liang et al. [35] method (Xception + LSTM). Moreover, our SC-MP-Mixer achieved 8.21% higher Acc than InceptionV3 + LSTM proposed by Liang et al. [35] 6.28% higher than ResNet50 + LSTM, and 7.08% higher than Xception + ResNet50 + LSTM. The SC-MP-Mixer method achieved 9.7% higher Acc than the CNN + SVM developed by Ekiz et al. [33], 7.73% higher than LeNet5 developed by Sharma et al. [26], and 4.87% higher than the Fused CNN presented by Banik et al. [27].

**Table 9:** Comparison classification results with different models in the literature

Study	Methodology	Dataset	Class	Acc (%)
Patil et al. [1]	CCA—(VGG16 + LSTM)	BCCD	4	89.85
Patil et al. [1]	CCA—(InceptionV3 + LSTM)	BCCD	4	91.06
Bani-Hani et al. [24]	CNN + Genetic Algorithm	BCCD	4	91.01
Liang et al. [35]	InceptionV3 + LSTM	BCCD	4	87.45
Liang et al. [35]	ResNet50 + LSTM	BCCD	4	89.38
Liang et al. [35]	Xception + LSTM	BCCD	4	90.79
Liang et al. [35]	Xception + ResNet50 + LSTM	BCCD	4	88.58
Ekiz et al. [33]	CNN + SVM	BCCD	4	85.96
Sharma et al. [26]	LeNet5	BCCD	4	87.93
Banik et al. [27]	Fused CNN	BCCD	4	90.79
Sharma et al. [29]	a fast traditional CNN	BCCD	4	84.64
Yildirim et al. [57]	GoogleNet, DenseNet, AlexNet, ResNet50 + Filters (Gauss and Median)	BCCD	4	75.21–83.44
Vatathanavaro et al. [58]	VGG16, ResNet50	BCCD	4	72.07–88.29
**Our method**	**SC-MP-Mixer**	BCCD	**4**	**95.66 ± 0.19**
Uçar et al. [30]	ShuffleNet	PBC	8	97.94
Acevedo et al. [50]	VGG16	PBC	8	96.00
Acevedo et al. [50]	InceptionV3	PBC	8	95.00
Long et al. [51]	Capsule network based model	PBC	8	99.3
Fırat [52]	Modified Inception Module	PBC	8	98.89
Atıcı et al. [53]	R-CNN Based Segmentation and Classification	PBC	8	99.31
**Our method**	**SC-MP-Mixer**	PBC	**8**	**99.65 ± 0.11**
Jiang et al. [45]	Discriminative region detection assisted feature aggregation network	Raabin	5	95.17
Akalin et al. [55]	The hybrid use of Detectron2 and YOLOv5	Raabin	5	98.00
Tsutsui et al. [54]	ViT-Base-16	Raabin	5	98.33
Tavakoli et al. [56]	SVM classifier	Raabin	5	94.65
**Our method**	**SC-MP-Mixer**	**Raabin**	**5**	**98.68 ± 0.17**

Bold indicates the best result

In the PBC dataset, our SC-MP-Mixer model achieved 99.65% Acc. Our SC-MP-Mixer model achieved 0.66% better results than the proposed method by Uçar et al. [30]. Similarly, it obtained 2.6% better accuracy than the proposed VGG16 method by Acevedo et al. [50] and 3.6% better than the InceptionV3 proposed by Acevedo et al. [50]. Moreover, our SC-MP-Mixer model achieved Acc values that were 0.35% higher than the Capsule network-based model suggested by Long et al. [51], 0.76% higher than the Modified Inception-based module developed by Fırat [52], and finally, 0.34% higher than the R-CNN-based classification presented by Atıcı et al. [53]. SC-MP-Mixer model achieved an Acc of 98.68% with the Raabin, which was another dataset used in the experimental studies. When compared to different studies using the Raabin, SC-MP-Mixer model shows the closest Acc value to our model at 98.33%, achieved by the ViT-Base-16 model developed by Tsutsui et al. [54]. However, our SC-MP-Mixer model outperformed the ViT-Base-16 model by 0.35% in Acc. Upon further examination of other models, our SC-MP-Mixer model obtained Acc values that were 3.51% higher than the Discriminative Region Detection Assisted Feature Aggregation Network model presented by Jiang et al. [45], 0.68% higher than the hybrid use of Detectron2 and YOLOv5 model developed by Akalin et al. [55], and 4.03% higher than the SVM used by Tavakoli et al. [56]. When all models in Table 9 are compared, it’s evident that SC-MP-Mixer model outperformed the studies in the literature across all three datasets, yielding significantly better results.

## Conclusions

In this study, a novel DL-based model is proposed for WBC classification. The proposed model is a new hybrid model based on ConvMixer and Swin transformer architectures. This hybrid model is called Multipath mixer (SC-MP-Mixer) based on Swin Transformer and ConvMixer. In our SC-MP-Mixer model, ConvMixer blocks extract features with strong spatial detail, while Swin transformer networks effectively handle these features with self-attention mechanism. Also, our SC-MP-Mixer model offers a multipath approach to get better patch representations for ConvMixer and Swin transformer blocks. In this approach, ConvMixer and Swin transformer blocks are applied in parallel with different patch sizes. In this way, more effective features can be obtained with different patch representations. Experimental studies were carried out on three different WBC datasets (BCCD, PBC and Raabin) to test the performance of the our SC-MP-Mixer model. These datasets are BCCD consisting of 4 classes (EO, L, M and N), PBC consisting of 8 classes (N, EO, B, L, M, IG, P, ER) and Raabin consisting of 5 classes (B, EO, L, M and N). Our SC-MP-Mixer method obtained 99.65% macro Acc, 98.71% F1s, 98.67% Pr, 98.76% Re with PBC, while 95.66% macro Acc, 91.44% F1s, 91.72% Pr, 91.33% Re with BCCD value has been obtained. In addition, 98.68% Acc, 94.42% F1s, 94.34% Pr and 94.63% Re values were obtained with the Raabin. Our SC-MP-Mixer model has been compared with the methods using these three datasets in the literature. As a result of the comparison, it was seen that our SC-MP-Mixer model achieved better classification results than other methods. These findings suggest that our SC-MP-Mixer model shows promise as an alternative approach in clinical experiments due to its capacity to efficiently and accurately extract WBC features for classification purposes. In future studies, the primary aim is to develop new models that will increase the accuracy values on the BCCD and Raabin datasets. Additionally, collaborations with expert physicians will involve work on private datasets, with plans to develop a software that will be made available for use by these expert doctors.

## Data Availability

Data will be made available on request.
